# Atypical Manifestations of Primary Pulmonary Adenocarcinoma With Signet Ring Features: A Case Report

**DOI:** 10.7759/cureus.35738

**Published:** 2023-03-03

**Authors:** Noah R Kosnik, Syed M Hasnain, Duncan A McKinney, Nelson Greene

**Affiliations:** 1 Internal Medicine, LewisGale Medical Center, Salem, USA; 2 Pulmonary and Critical Care, LewisGale Medical Center, Salem, USA

**Keywords:** bronchoscopic biopsy, mediastinal lymphadenopathy, pulmonary signet ring adenocarcinoma, tracheobronchial polyps, primary pulmonary adenocarcinoma, secondary tracheobronchial tumors

## Abstract

Primary pulmonary malignancies are one of the most common malignancies worldwide. The most common non-small cell lung malignancy is adenocarcinoma, but there are many subtypes with different molecular and genetic expressions causing different manifestations. Uncommon manifestations include persistent back pain and tracheal bronchial tumors. More than 95% of reported tracheal bronchial tumors are benign and thus are rarely biopsied. There are no reported cases of secondary tracheal bronchial tumors due to pulmonary adenocarcinoma. Today, we are reporting the first case report of an uncommon manifestation of primary pulmonary adenocarcinoma.

## Introduction

Manifestations of primary lung malignancy range from incidental detection in asymptomatic individuals to severe in the case of multiorgan metastasis. Pulmonary adenocarcinoma is no different in that it can present in a variety of manners. Incidental discovery of lung nodules on imaging is a common presentation for the asymptomatic and those with subclinical symptoms.

As pulmonary cancer progresses, common complaints include cough (nonproductive to hemoptysis), dyspnea, chest pain, and vocal changes. Alternatively, atypical cases may manifest with back pain, superior vena cava (SVC) syndrome, Horner syndrome, or tumors discovered on bronchoscopy [1]. 

Primary pulmonary cancers are among the most common malignancies worldwide and are only surpassed in the United States by breast and prostate cancer in women and men, respectively [2]. Pulmonary malignancies are classified into the small cell (15%) and non-small cell (85%) based on histologic appearance, with the most common non-small cell subtypes consisting of adenocarcinoma (~43%), squamous cell carcinoma (~25%), and large cell carcinoma (~17%) [3]. Pulmonary adenocarcinoma is further categorized into subtypes based on general appearance as well as immunochemical and pathologic factors [4]. The case presented in this study will describe an atypical manifestation of primary pulmonary adenocarcinoma with signet ring features.

## Case presentation

A 46-year-old African American female with a medical history of chronic sinusitis, vitamin D deficiency, and chronic tobacco use presented to the emergency room with a chief complaint of shortness of breath (SOB). Her SOB was noted one month prior and progressed over that period in terms of frequency and intensity. Initially, her symptoms improved with rest; however, by the time she presented for evaluation, she endorsed persistent dyspnea unrelieved by rest. No supplemental oxygen was utilized by the patient throughout this period.

Around the time that she noticed SOB, she experienced concurrent back pain centered around the midline that progressed laterally along her right upper back. A productive cough was described as a clear consistency without hemoptysis. She did not have a primary care provider or pulmonologist before this episode and had not seen a healthcare provider for multiple years. She endorsed loss of appetite, night sweats, and loss of 30 lbs over the month prior.

**Figure 1:**
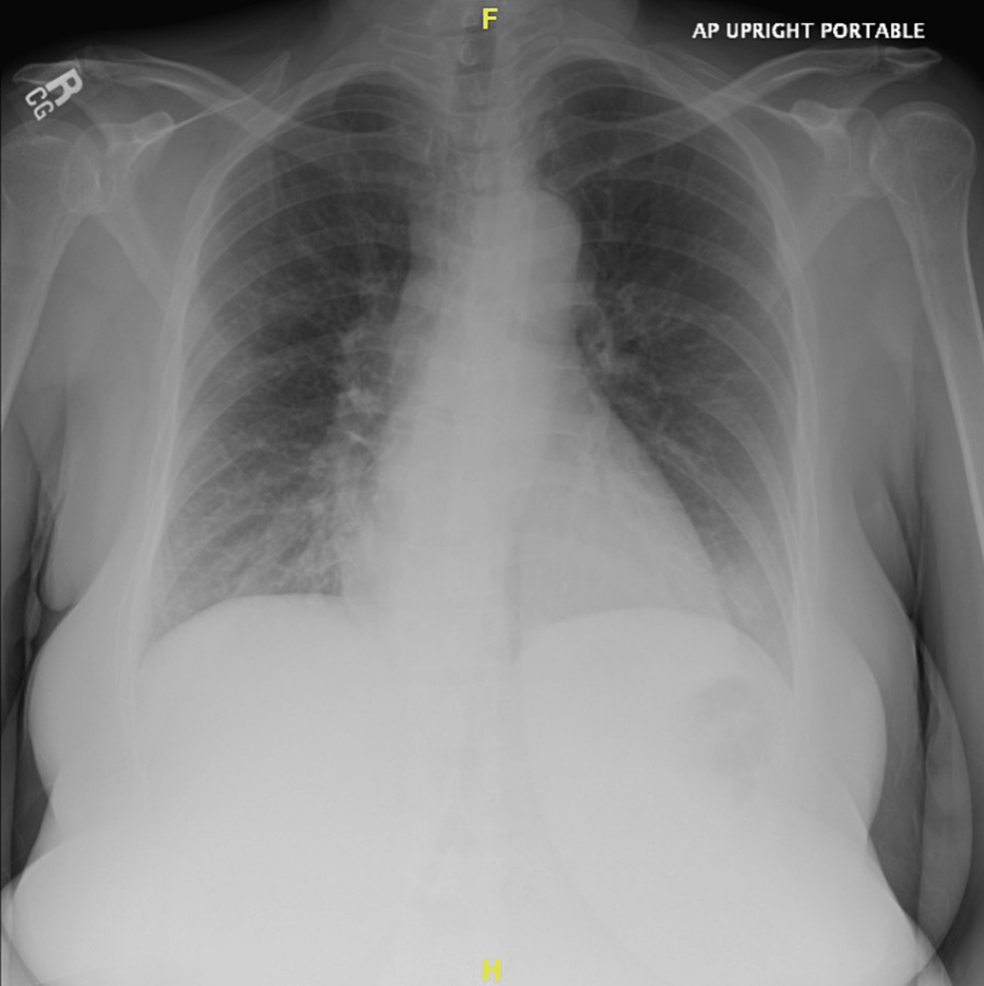
A/P upright chest X-ray: normal cardiomediastinal borders in size and contour and negative for pneumonia, pneumothorax, or pleural effusion. A/P, anteroposterior

**Figure 2:**
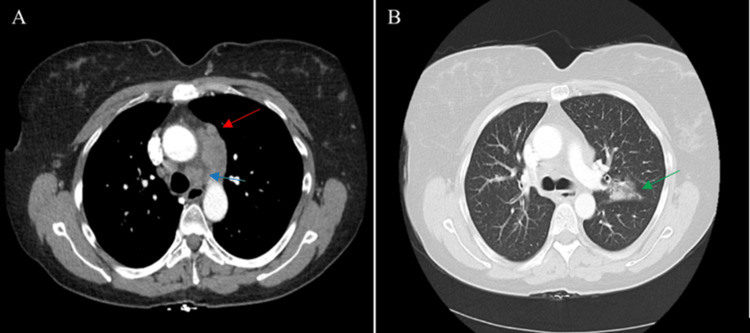
(A) A transverse CT of the chest showing the preaortic lymph node (red arrow) and 4.8 cm × 2.6 cm mediastinal lymphadenopathy (blue arrow) included in the AP window node (no hilar or axillary adenopathy); (B) transverse CT within the lung window of the chest showing a ground-glass consolidation 2.9 cm × 2.1 cm lesion in the left upper lobe (green arrow). AP, aortopulmonary; CT, computed tomography

In the emergency department, a chest X-ray revealed no acute cardiopulmonary disease (Figure 1). High suspicion by emergency medical staff for pulmonary embolism resulted in a computed tomography angiography (CTA) of the chest. Imaging showed mediastinal lymphadenopathy, a left upper lobe ground-glass consolidation, and multiple scattered sclerotic lesions throughout the thoracic spine (Figures 2A-2B). The patient was hospitalized for further workup and pain management.

**Figure 3:**
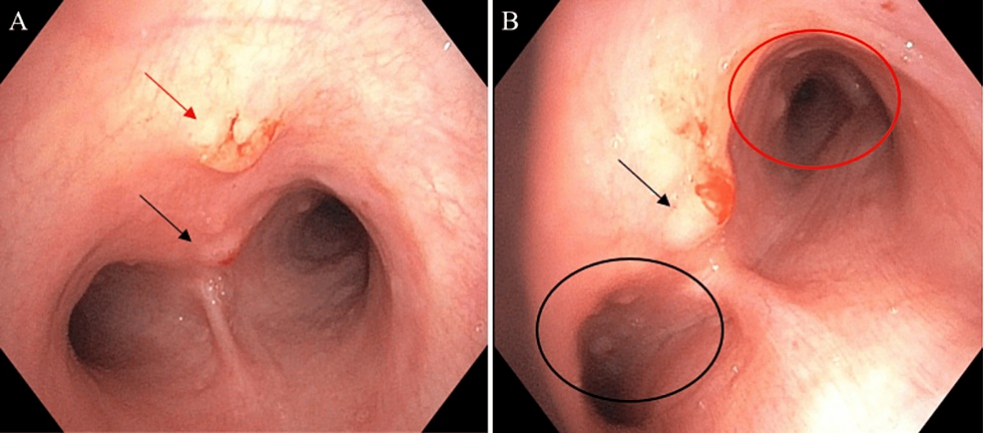
(A) Visualization of the airway in the subglottic and trachea regions using flexible bronchoscopy and anterior proximal tracheal polyp (red arrow) with a distal tracheal polyp (black arrow) around the carinal bifurcation; (B) visualization of tracheal polyp proximal to the carina (black arrow) with multiple smaller polyps in the left mainstem bronchus (black circle) and multiple larger polyps (red circle) in the right mainstem bronchus.

Concern for lung cancer with metastasis to bone was discussed with the patient, and a diagnostic flexible bronchoscopy with ultrasound-guided lymph node biopsy to further identify the pulmonary lesion was performed. During bronchoscopy, incidental lesions along the anterior trachea were noted and thought to be benign (Figure 3A). Distal movement of the flexible bronchoscope revealed multiple polyps along the left and right mainstem bronchi (Figure 3B). Biopsies of the midline anterior tracheal polyp and supracarinal polyp were obtained and sent for pathological analysis. Ultrasound-guided lymph node biopsies of stations 4R, 4L, 7, 11S, 10R, and 11L were also obtained for pathological analysis.

A follow-up pathology report was obtained seven days after bronchoscopy and showed invasive adenocarcinoma with signet ring features. The lymph node and polyp tissues were sent for genetic analysis and showed positive protein expression for cytokeratin 7 (CK7), napsin A, and thyroid transcription factor-1 (TTF-1) and negative protein expression for CDX2, CD56, synaptophysin, and CK20.

The patient was diagnosed with primary pulmonary adenocarcinoma with secondary metastasis to the mainstem bronchi and trachea. Further workup including a positron emission tomography (PET) scan was scheduled to be performed in an outpatient setting.

## Discussion

Both the prevalence of lung cancer and its association with increased potential for poor outcomes have sustained investigation into the study and classification of pulmonary malignancy. Primary pulmonary cancer rates collected from 2020 showed a frequency of 1.8 million cases worldwide, including 235,000 in the United States [2]. Mortality from pulmonary cancers was estimated to account for 21.4% of all cancer-related deaths in the United States, with a five-year relative survival rate of 22.9% in 2022 [5].

Adenocarcinoma now accounts for nearly half of all lung cancers, and the relative frequency continues to increase. This change comes in contrast with the static prevalence of others, as well as the decreasing frequency of squamous cell carcinoma of the lung. These trends are hypothesized to reflect patterns of tobacco use (as squamous cell carcinoma has a higher correlation with tobacco abuse than any other non-small cell lung cancer [NSCLC]); however, this is yet to be definitively proven [2].

Advances in the understanding of genetic and biomolecular underpinnings, histologic appearances, and pathophysiologic patterns of cancer have necessitated revisions to the methods of oncologic description and categorization. The accepted definitions of individual pulmonary malignancies have been addressed frequently over the last decade, with revisions to the World Health Organization (WHO) classification guidelines occurring in both 2015 and 2021. The 2015 revision focused on the utilization of advanced immunohistochemical techniques for classification, while the 2021 updates instead relied upon molecular pathology and genetic testing [6]. Based on changes made to the 2015 WHO census, the subtypes of clear cell and signet ring adenocarcinoma were discontinued and recognized as a feature when any amount is present under pathological evaluation (WHO) [7].

Primary pulmonary adenocarcinoma with signet ring features is a rare finding unto itself, but in this instance, the presentation of atypical phenotypic manifestations along with the discovery of numerous tracheal and bronchial polyps makes for an excellent teaching case. Isolated tracheobronchial tumors (TBTs) are an exceptionally rare finding and account for approximately 0.6% of all pulmonary cancers [8]. Primary benign TBTs are thought to account for only 9.4% of tumors, while metastatic tumors to the tracheobronchial area account for 90.6% [9]. Thus, when nodules are discovered on bronchoscopy, a thorough multisystem evaluation is warranted. For the case described above, it is speculated that the ground-glass lesion found in the left upper lobe may represent a primary site that metastasized to the mediastinum and the tracheobronchial tract and spine from there (Figure 2b).

Once a TBT is biopsied, histologic and molecular analysis can assist with determining the location of the primary lesion should there be one. Histologically, versions of pulmonary adenocarcinoma and gastrointestinal tumors can share similar histological patterns, including mucinous and signet rings. The molecular expression can assist in the differentiation of its origin. Common molecular markers that indicate pulmonary etiology versus gastrointestinal tumors are CK7, CK20, CDX2, and TTF-1. Primary pulmonary signet ring tumors will react strongly only with CK7 and TTF-1, whereas metastatic gastrointestinal lesions will be positive for CK20 and CDX2 [4].

Pulmonary malignancies are often evaluated and treated aggressively based on their tendency for poor outcomes and potential for metastasis. When TBTs are discovered, there should be a high degree of suspicion for metastasis (either from or to additional sites) as these variants are highly associated with malignancy. A multidisciplinary approach to the diagnosis and management of these cases is crucial. Diagnostic testing should include extensive imaging via radiology and sample collection with endoscopic bronchoscopy biopsy for analysis. Specialists from pulmonology, thoracic surgery, oncology, radiology, pathology, and anesthesiology may be required for the management.

## Conclusions

Physical manifestations of primary lung malignancy have a vast array of phenotypes. In patients who have a high suspicion of pulmonary malignancy and present with benign-looking lesions within the airway, it is recommended that a biopsy of the lesion be obtained for pathological analysis.
